# Developing a group intervention to manage fatigue in rheumatoid arthritis through modifying physical activity

**DOI:** 10.1186/s12891-019-2558-4

**Published:** 2019-05-04

**Authors:** Victoria E. Salmon, Sarah Hewlett, Nicola E. Walsh, John R. Kirwan, Maria Morris, Marie Urban, Fiona Cramp

**Affiliations:** 1 Institute of Health Research, University of Exeter College of Medicine and Health, College House, St Luke’s Campus, Heavitree Road, Exeter, EX1 2LU UK; 2 Faculty of Health & Applied Sciences, University of the West of England, Blackberry Hill, Bristol, BS16 1DD UK; 3 Academic Rheumatology, University of Bristol, University of Bristol, Senate House, Tyndall Avenue, Bristol, BS8 1TH UK; 4 Bristol Royal Infirmary, University Hospitals Bristol NHS Foundation Trust, Upper Maudlin St, Bristol, BS2 8HW UK

**Keywords:** Fatigue, Rheumatoid arthritis, Physical activity, Intervention development, Self-management, Patient and public involvement, Co-design

## Abstract

**Background:**

Fatigue is a major symptom of rheumatoid arthritis (RA). There is some evidence that physical activity (PA) may be effective in reducing RA fatigue. However, few PA interventions have been designed to manage fatigue and there is limited evidence of end-user input into intervention development. The aim of this research was to co-design an intervention to support self-management of RA fatigue through modifying PA.

**Methods:**

A series of studies used mixed methodological approaches to co-design a fatigue management intervention focused on modifying PA based on UK Medical Research Council guidance, and informed by the Behaviour Change Wheel theoretical framework. Development was based on existing evidence, preferences of RA patients and rheumatology healthcare professionals, and practical issues regarding intervention format, content and implementation.

**Results:**

The resulting group-based intervention consists of seven sessions delivered by a physiotherapist over 12 weeks. Each session includes an education and discussion session followed by supervised PA chosen by the participant. The intervention is designed to support modification and maintenance of PA as a means of managing fatigue. This is underpinned by evidence-based behaviour change techniques that might support changes in PA behaviour. Intervention delivery is interactive and aims to enhance capability, opportunity and motivation for PA.

**Conclusion:**

This study outlines stages in the systematic development of a theory-based intervention designed through consultation with RA patients and healthcare professionals to reduce the impact of RA fatigue. The feasibility of future evaluation of the intervention should now be determined.

## Background

Fatigue is an important symptom of rheumatoid arthritis (RA) [1]. Patients report fatigue as difficult to manage with little professional support [2, 3]. The multi-dimensional nature of RA fatigue has been largely ignored, with little treatment targeted specifically at this symptom [4]. Despite inclusion of fatigue measurement in clinical trials [5], few studies have explicitly addressed RA fatigue management.

RA fatigue has been associated with reduced participation in physical activity (PA) [6, 7]. However, meta-analyses suggest that PA may have a small beneficial effect on RA fatigue [8, 9]. To date, few studies have investigated interventions designed specifically to reduce fatigue, or examined fatigue as a primary outcome. The need to develop a specific PA fatigue management intervention that meets the needs of RA patients is evident.

Designing complex interventions to improve health outcomes requires systematic development to ensure they are likely to be worth implementing in clinical practice [10]. Complex interventions have several dimensions, including the number and difficulty of behaviours required by recipients and those delivering the intervention and the permissible degree of flexibility or tailoring of the intervention [10]. Involving stakeholders in a co-design process enables their experiences to be incorporated into the intervention, increasing the likelihood of it being acceptable to users and providers [11]. Using a co-design approach provides an opportunity to explore challenges, concerns and ideas for future implementation at an early stage, improving potential effectiveness and enhancing uptake, adoption and maintenance of the intervention.

UK Medical Research Council (MRC) guidance for developing complex interventions recommends three processes in early development:

1. identifying an existing evidence base
2. identifying/developing appropriate theory
3. modelling processes and outcomes [10]

Whilst MRC guidelines strongly advocate a theoretical basis, they do not provide detailed guidance on how to choose or apply theory. In recognition of this, the Behaviour Change Wheel (BCW) has been proposed as a comprehensive, systematic approach to intervention development based on established behaviour change theory [12]. The central behaviour system in the BCW is the theoretically-based Capability, Opportunity, Motivation – Behaviour (COM-B) model, that suggests a change in behaviour will require a modification in at least one of the following components: the ‘capability’ of a person to carry out that behaviour; the ‘opportunity’ for the behaviour to occur; and ‘motivation’ to perform the behaviour at that moment in time [12].

The BCW framework assists intervention developers in identifying potential concepts required for behaviour change, as well as aiding designers in analysing target behaviours and characterising interventions and their active components. It is supported by links to other theory-based resources, such as the Theoretical Domains Framework (TDF) [13] and a taxonomy of recognised behaviour change techniques (BCTs) [14]. It is recommended in UK National Institute for Health and Care Excellence (NICE) guidance for developing individual-level behaviour change interventions [15].

Few studies describe a collaborative co-design process for the development of PA interventions in RA [16, 17]. Despite recommendations for the use of theory in self-management interventions in rheumatology [18], few studies report how theory was used during development of PA interventions for RA [19, 20]. The aim of this research was to co-design an intervention with RA patients and healthcare professionals (HCPs) to support self-management of RA fatigue through modifying PA. Intervention development was guided by the MRC framework and based on existing evidence, RA patient and HCP preferences and priorities, underpinned by the BCW theoretical framework. The objectives were to 1) develop programme content; 2) design programme sessions and develop resources required for delivery; and 3) develop educational support materials.

## Methods

**Fig. 1:**
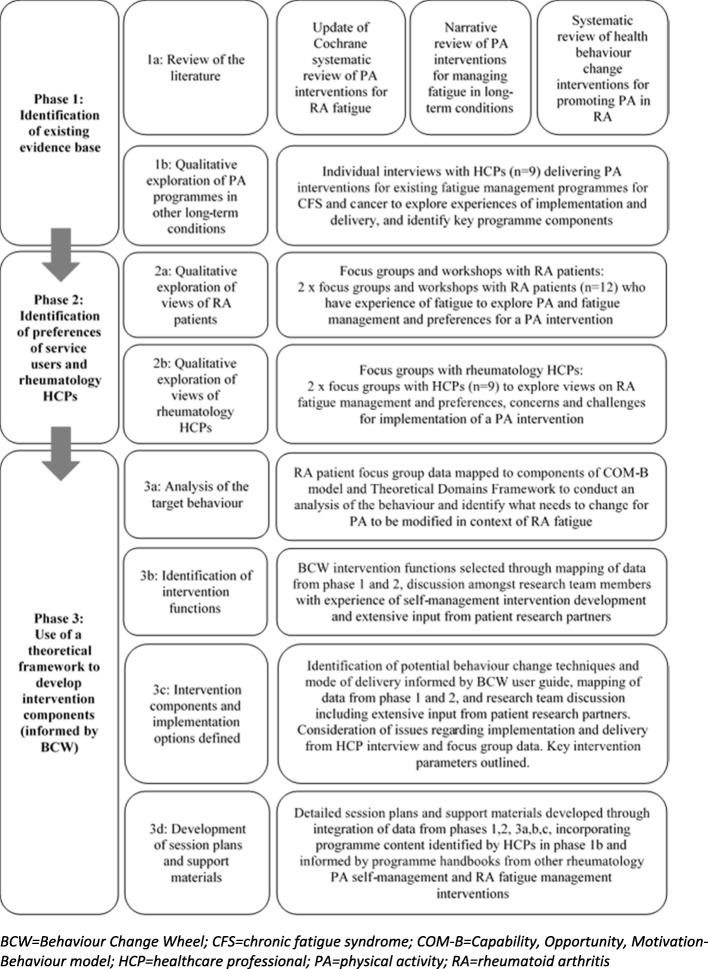
Intervention development process

Full methods are available elsewhere [21]. Intervention development (Fig. 1) was supported by a research team and patient research partners (PRPs) with experience of developing theory-based self-management interventions for rheumatic diseases. The multi-disciplinary research team had professional backgrounds in physiotherapy (VS, FC, NW), rheumatology nursing (SH) and rheumatology medicine (JK). Both PRPs (MM, MU) had a diagnosis of RA and had experienced fatigue, providing a perspective of the lived experience. PRP involvement in research improves research quality [22], with benefits including a fresh perspective, changes to study designs and novel outcomes [23].

### Phase 1: identification of an existing evidence base

#### 1a: review of the literature

Three literature reviews were conducted to 1) identify existing evidence and investigate the effectiveness of PA interventions for reducing RA fatigue; 2) identify existing evidence for the effectiveness of PA and exercise therapy interventions for fatigue in other conditions, and describe intervention characteristics and methods of delivery; and 3) evaluate the effectiveness of interventions incorporating health behaviour change theory or techniques for PA promotion, uptake and maintenance in RA.

#### Search methods

Reviews 1 and 3 used Cochrane systematic review methodology [24]. Review 1 was based on a Cochrane review investigating non-pharmacological interventions for managing RA fatigue [8]. Review 2 used systematic searching to identify papers for a narrative review. Electronic databases were searched up to March 2015: MEDLINE, Allied and Complementary Medicine (AMED), Cumulative Index to Nursing and Allied Health Literature (CINAHL) Plus, Cochrane Controlled Trials Register (CENTRAL), EMBASE, PsycINFO, SportDiscus, Science Citation Index.

#### 1b: qualitative exploration of PA programmes in other long-term conditions

Semi-structured interviews were conducted with HCPs (*n* = 9) delivering PA interventions for fatigue management in cancer or chronic fatigue syndrome (CFS). The University of the West of England Faculty of Health and Applied Sciences Research Ethics Committee approved the study (Ref: HLS/12/11/139). Interviews explored opinions and experiences of HCPs, key components of the PA content of programmes and use of health behaviour change theory and/or BCTs.

#### Data analysis

Discussions were audio-recorded and transcribed verbatim. Interviews were analysed (VS) using hybrid thematic analysis [25, 26], using a BCT taxonomy [27] as a deductive framework. A subset of transcripts was independently analysed (SH, MU).

### Phase 2: identification of preferences of RA patients and rheumatology HCPs

Phase 2 was approved by the National Research Ethics Service Committee East Midlands - Nottingham 1 (Ref: 13/EM/0331).

#### 2a: qualitative exploration of preferences of RA patients

Two focus groups were conducted with purposively selected RA patients (*n* = 12) who self-reported RA fatigue. The acceptability of a PA intervention for managing RA fatigue was explored.

Each group participated in a workshop to identify preferences for intervention structure, delivery and content. Multiple-choice questions identified from phase 1b were presented and participants voted for the response that they most agreed with using real-time data collection, providing instant feedback to participants. Individual responses remained anonymous. Responses were collected via TurningPoint handsets and imported into Microsoft Excel (2007). Participants discussed their views regarding the presented content, such as which components would be most useful and acceptable as part of an RA fatigue management intervention. Comments were recorded in field notes.

#### 2b: qualitative exploration of views of rheumatology HCPs

Data from phase 2a were collated and summarised prior to two further focus groups with rheumatology HCPs (*n* = 9). Participants discussed challenges, opportunities and practical considerations regarding implementation of a PA fatigue management intervention.

#### Data analysis

Focus groups were audio recorded, transcribed verbatim and analysed (VS) using inductive thematic analysis [25]. Transcripts were independently analysed (FC, NW, MM). All analyses were discussed and agreed with the research team. Workshop data were collected via TurningPoint (TurningPoint version 4.2.3, www.turningtechnologies.com) and analysed in Microsoft Excel (2007).

#### Phase 3: use of a theoretical framework to develop intervention components

The BCW guide to designing interventions was used to develop intervention components [28]. Data from phases 1 and 2 were mapped onto the theoretical framework in order to:

- Understand the behaviour (3a)
- Identify intervention options (3b)
- Identify content and implementation options (3c)

Session plans and support materials were developed (phase 3d) based on outcomes from phases 3a-3c.

#### 3a: analysis of target behaviour

Qualitative data from phases 1b, 2a and 2b were mapped onto domains of the COM-B model and TDF. This developed a comprehensive theoretical understanding of what might need to change for RA patients to modify PA as a means of managing their fatigue.

#### 3b: identify intervention functions

Intervention functions that might bring about a change in behaviour were selected according to evidence of effectiveness for the given situation and population; relevance to the target behaviour, setting and population; feasibility of delivery of the function; acceptability to patients and professionals [28]. Where possible these criteria were supported by evidence from phases 1 and 2. Where these data were not available selection was made through discussion with the research team.

#### 3c: identify content and implementation options

Selection of intervention content was guided by data from phase 1 and 2 and informed by programme manuals provided in phase 1b, from existing evidence-based interventions in CFS [29] and for current research trials for a PA self-management intervention for chronic pain [30] and a cognitive behaviour therapy-based RA fatigue self-management intervention [31].

Identification and selection of potential BCTs to deliver intervention functions and decisions regarding mode of delivery were informed by the BCW guide [28], BCT taxonomy [14], mapping of phase 1 and 2 data, and research team discussions. Core BCTs were selected from those recommended for inclusion in PA programmes [32, 33] and additional BCTs identified from phase 1b. The criteria of effectiveness, relevance, feasibility and acceptability used to select functions were applied when choosing BCTs. Issues regarding implementation and delivery identified in phases 1b and 2 were considered.

#### 3d: development of session plans and support materials

Theory-informed content and delivery options were combined with patient and rheumatology HCP preferences and practical issues to produce a draft intervention. Individual session plans were developed along with educational support materials. Further decisions about who should deliver the intervention, intervention format and setting, session frequency and duration were considered.

The research team reviewed individual session plans and accompanying support materials. In-depth discussions were held with PRPs to check the order of session topics and to ensure that content and materials were readable, comprehensive and useful. Comments provided by all team members were used to amend and refine the intervention.

## Results

### Participant characteristics

Eighteen HCPs (all female) consented to take part in phases 1b (*n* = 9) and 2b (n = 9). They had been qualified between 11and 32 years (phase 1b) and 7–28 years (phase 2b) with rheumatology experience ranging from 5 to 15 years for phase 2b participants. Most HCPs were physiotherapists (*n* = 12) with two occupational therapists also participating in both phases. In addition, in phase 1b a clinical nurse specialist and exercise physiologist participated.

Twelve patients (6 female), aged 43–66 years (mean 56.8) with disease duration 0.25–25 years (mean 8.2), consented to participate in phase 2a.

### Phase 1: identification of an existing evidence base

#### 1a: review of the literature

Review 1 findings have been published elsewhere [34]. Only two additional studies were identified since the Cochrane review [8]. The original meta-analysis conclusion that there was a small beneficial short-term effect of PA for managing RA fatigue remained unaltered.

Review 2 concluded that aerobic PA using a graded approach might be particularly effective for managing fatigue. Optimal intervention parameters, such as duration, frequency and intensity of PA were unclear, although some evidence suggested incremental increases from a lower intensity of PA might yield better outcomes in CFS [35]. There were limited data regarding adherence to treatment and research procedures and no qualitative studies were identified exploring acceptability of PA for fatigue in other long-term conditions.

Review 3 highlighted a lack of evidence for the effectiveness of theory-based interventions to promote engagement in and long-term maintenance of PA in RA. Interventions that promoted PA in RA employed a range of BCTs, including instruction on how to perform the behaviour, information provision, goal setting, problem-solving, feedback and self-monitoring. Although some studies specified or alluded to health behaviour change theories, it was unclear if or how theory was used during intervention development.

#### 1b: qualitative exploration of PA programmes in other long-term conditions

Key themes from interviews with HCPs (*n* = 9) related to format and delivery of PA interventions, and the need for organisational flexibility.

#### Format

HCPs (n = 9) identified various approaches to providing PA interventions for fatigue management in long-term conditions, including group and individual programmes of variable length. All programmes were delivered face-to-face with differences in the number, frequency and duration of sessions. Consistent findings included the use of a graded approach to PA and a need to address psychosocial and motivation issues relating to PA and fatigue. Several BCTs were identified, including instruction on performing PA, demonstration of PA, encouraging rehearsal of PA and delivery by a credible source. However there was inconsistency in the application of techniques clinically. Some participants mentioned cognitive behavioural approaches, but these were not firmly embedded within existing programmes.

#### Delivery

HCPs advocated a patient-centred, interactive approach to delivery, with emphasis on patient-led problem solving. Most participants tried not to be prescriptive regarding PA, allowing patients to choose activities that were relevant and appealing. Motivational interviewing (MI) [36] was considered useful to improve engagement with PA.

#### Organisational flexibility

Organisational flexibility was required to ensure accessibility, meet training needs of staff, evaluate programmes and measure outcomes. Location and timing of sessions were considered important. For example, fatigued patients might struggle to attend morning sessions or travel long distances.

### Phase 2: identification of preferences of RA patients and rheumatology HCPs

All phase 2 participants supported the use of PA for managing RA fatigue. Key themes relating to challenges and solutions for implementation concerned group and peer support, patient-centred delivery, accessibility and knowledge and skills.

#### Group and peer support

All participants felt that PA interventions should be delivered face-to-face in groups. Patients considered groups extremely valuable, providing an opportunity for interaction and discussion with fellow patients, as well as HCPs, who could offer expert advice and reassurance, enhancing confidence with managing fatigue and modifying PA. They felt that a group format was key for problem solving and learning through peer support. HCP participants agreed that group sessions had the advantage of offering peer support and felt they could be justified from a management perspective. However, lack of staff and limited resources may prohibit group sessions in some services.

#### Patient-centred delivery

Patient participants felt that consideration of patient preferences and availability of choice was crucial to enhance motivation. While some HCPs had concerns about managing patient choice in a group setting, others agreed that choice was important for patients. This echoed phase 1b findings that patient choice and decision-making are crucial for a successful outcome. Some phase 2b participants believed that delivery style was key to a successful intervention, reporting that MI techniques could be useful in this regard. Both patients and HCPs supported inclusion of a practical PA component to enhance confidence with PA in a supportive environment. All participants agreed that PA should be tailored to individual needs, with a choice of exercises to accommodate level of ability.

#### Accessibility

Patient participants indicated that attending morning sessions is often difficult, as fatigue and other RA symptoms are often worse at this time of day, confirming findings in phase 1b. Patients in paid employment, or with caring roles and responsibilities raised concerns regarding limited access to services during working hours. Provision of out-of-hours services was challenging for HCPs, although some phase 2b participants reported positive experiences of flexible staffing, providing opportunities for delivering evening or weekend sessions.

Patient preferences for location varied between community and hospital settings. Participants recruited from an inner-city hospital indicated that available transport options may influence their decision to attend a programme.

Not all HCPs had access to premises that would allow a group of 6 to 10 patients to be seated comfortably for the discussion session, or access to an appropriate space for a practical PA session. These issues are likely to differ according to local circumstances and would need to be addressed prior to implementation.

Duration and frequency of sessions and programme length were discussed. Patients indicated a preference for sessions of up to 2 h in duration, delivered over 12 to 14 weeks. Despite some concerns, HCP participants with experience of delivering 2-h sessions found these were well received by patients. Most HCPs had concerns about delivering a programme of more than 6 weeks. However, it was felt that this might be achievable by adjusting session frequency, believing that this could promote independence with self-management and PA, and improve long-term adherence.

#### Knowledge and skills

Patients identified a lack of knowledge and skills to self-manage their fatigue and PA. Those who had received HCP support with self-management skills, such as analysing and interpreting activity patterns using activity diaries, had found this extremely useful. Patients wanted expert advice regarding PA and fatigue management, suggesting that someone with good knowledge of RA, fatigue and PA should lead the intervention. This could be a HCP or a trained exercise professional. HCPs and patients agreed that techniques that enable patients to problem-solve challenges and identify opportunities for modifying PA should be incorporated in an intervention.

HCPs believed that implementation of a PA intervention may require additional training in fatigue management, graded approaches to PA and/or basic psychosocial skills, depending on experience and knowledge. In addition, HCP participants indicated that delivery of fatigue management programmes by physiotherapists would require a change in referral practices, as current fatigue advice is usually provided by occupational therapists.

### Phase 3: use of a theoretical framework to develop intervention components

#### 3a: analysis of the target behaviour

The target group for this intervention were adults with RA who experience fatigue. The behavioural target was modification of PA in daily life. The behavioural analysis indicated that there was a potential need for change in all components of the COM-B model in order for RA patients to modify PA within the context of fatigue (see Table 1 for example, and supplementary file 1 for full tables).

**Table 1:** Example behavioural analysis for reflective motivation component of COM-B

COM-B	Theoretical domain	What needs to happen for the target behaviour to occur?	Example evidence of need for change or support for inclusion
Reflective motivation	Professional/social role and identity	Encourage being active as part of identity	**011: “it’s a long ongoing battle where you’ve had to forget your old life, what you used to do, I’ve given up work and stayed home.”** **015: “… [RA] it’s changed my life, I get depression. I have changed, I am not the same person I was 3 years ago. I can’t talk for anybody else, but I’ve changed, I know I have.”**
	Beliefs about capabilities	Explore acceptance of having RA and fatigue and its effect on ability Address confidence with PA Identify PA that feel capable of doing, that is achievable	**014: “You can’t work to a regime of fitness because you never know what you’re allowed to do the next day.”** **016: “Mine’s quite bad in my joints. I’ve got chronic in my shoulders, in my neck, my feet, my hands, that’s it. I’ve got bare movement so I can’t do nothing.”**
	Optimism	Explore confidence with achieving PA goals	**011: “The minute you mentally make yourself kitted up ready to do it and then you fail at the first hurdle.”** **013: “And it’s horrible failing.”**
	Beliefs about consequences	Address beliefs about the effects of PA on fatigue and general consequences of PA Encourage belief that managing PA will have positive benefits for managing fatigue Address negative beliefs	**011: “I think physical activity does increase your fatigue, but also, on the other foot, decrease it as well.”** **021: “… I tried swimming and it caused flares in my shoulders. So I went to see the doctor about it and they said try an exercise bike for a minute a day, and that used to set off in my knees.”**
s	Intentions	Explore plans/intentions to be more active or to manage PA Encourage formulation of plans to carry out PA and implementation of specific PA goals Address setbacks and potential barriers to PA	**011: “Just like getting up and thinking, “Right, am I going to be able to do this today,” to try and do that and get myself to the swimming pool. Then the minute you get out of bed you collapse because you can’t put your foot to the floor”** 007:“if a patient comes in and they’ve had a sudden setback […] we would look at where they are in that setback, look at setback planning and how to think about and learn from that setback.”
	Goals	Explore expectations and desired achievements Set specific goals for PA	*024: “…finding out what their goals are and working towards them, and building their confidence with that.”*

*COM-B=Capability, opportunity, motivation, behaviour; GP = general practitioner; PA = physical activity; RA = rheumatoid arthritis*

Underlined text = quotations from healthcare professionals in interviews (phase 1b)

**Bold text = quotations from RA patients in focus groups (phase 2a)**

*Italic text = quotations from rheumatology healthcare professionals in focus groups (phase 2b)*

#### 3b: identification of intervention functions

Six intervention functions were selected. Education, persuasion, incentivisation, training and enablement have been linked to effective BCTs for increasing PA [12]. Modelling was also included, as phase 1b participants noted the importance of vicarious learning, and demonstration of the behaviour was considered a useful BCT.

#### 3c: intervention components and implementation options defined

Selected content, mode of delivery, core BCTs and intervention functions were mapped onto COM-B and TDF components (see Table 2 for example, and supplementary file for full version).

**Table 2:** Example mapping of COM-B reflective motivation component and TDF domains to intervention content, mode of delivery, core BCTs and intervention functions

COM-B	Theoretical domain from TDF	What needs to happen for the target behaviour to occur?	Content	Mode of delivery	Core BCTs	Intervention function
Reflective motivation	Professional/social role and identity	Encourage being active as part of identity	Review and feedback on progress Identify personal benefits of PA for RA and fatigue	Interactive group education and discussion Practical PA session with support from course leader Homework tasks, e.g. implementation of chosen PA between sessions	1.1, 1.5, 2.2, 3.1, 5.1, 8.1, 8.6	Education, Persuasion, Modelling
	Beliefs about capabilities	Explore acceptance of having RA and fatigue and its effect on ability Address confidence with PA Identify PA that feel capable of doing, that is achievable	Explore current feelings and experiences relating to fatigue and PA Participate in chosen PA Review and feedback on progress	Interactive group education and discussion Practical PA session with support from course leader Facilitated individual goal setting during practical PA session Educational support materials, e.g. handouts: goal setting, list of exercises included in practical session, monitoring PA, activity diary, pedometer Homework tasks, e.g. goal setting task, implementation of chosen PA between sessions	1.1, 1.2, 2.2, 2.3, 2.4, 4.1, 8.1, 8.6, 8.7	Education, Persuasion, Modelling, Enablement
	Optimism	Explore confidence with achieving PA goals	Setting individual goals for PA Review and feedback on progress Participate in chosen PA	Interactive group education and discussion Practical PA session with support from course leader Facilitated individual goal setting during practical PA session Educational support materials, e.g. handouts: goal setting, list of exercises included in practical session Homework tasks, e.g. goal setting task, implementation of chosen PA between sessions	1.1, 1.5, 1.6, 2.2, 2.3, 2.6, 8.1, 8.6	Education, Persuasion, Modelling, Enablement
	Beliefs about consequences	Address beliefs about the effects of PA on fatigue and general consequences of PA Encourage belief that managing PA will have positive benefits for managing fatigue Address negative beliefs	Identify benefits of PA for RA and fatigue Introduce and apply principles of graded approaches to PA Participate in chosen PA	Interactive group education and discussion Practical PA session with support from course leader Educational support materials, e.g. handouts: PA in RA, graded approaches to PA, list of exercises included in practical session Homework tasks, e.g. implementation of chosen PA between sessions	2.3, 2.4, 4.3, 5.1, 5.6, 8.1, 8.7, 9.1	Education, Persuasion, Modelling
	Intentions	Explore plans/intentions to be more active or to manage PA Encourage formulation of plans to carry out PA and implementation of specific PA goals Address setbacks and potential barriers to PA	Set individual goals for PA Review and feedback on progress Explore barriers and opportunities for implementing PA Devise plans for managing setbacks Identify opportunities for continuing and maintaining PA in the longer term	Interactive group education and discussion Facilitated individual goal setting during practical PA session Educational support materials, e.g. handouts: goals setting, graded approaches to PA, monitoring PA, managing setbacks Homework tasks, e.g. goal setting task, complete graded PA plan, formulate setback plan, implementation of chosen PA between sessions	1.1, 1.2, 1.4, 1.5, 1.6, 2.2, 2.3, 3.1	Education, Persuasion, Incentivisation, Modelling
	Goals	Explore expectations and desired achievements Set specific goals for PA	Set individual goals for PA Review and feedback on progress	Interactive group education and discussion Facilitated individual goal setting during practical PA session Educational support materials, e.g. handouts: goals setting, graded approaches to PA, monitoring PA, managing setbacks Homework tasks, e.g. goal setting task	1.1, 1.4, 1.6, 2.2, 2.3, 2.4	Education, Persuasion, Incentivisation, Modelling

*BCT = behaviour change technique; COM-B=Capability, Opportunity, Motivation, Behaviour; PA = physical activity; RA = rheumatoid arthritis; TDF = theoretical domains framework*

*BCT codes (from BCT taxonomy version 1* [14]*): 1.1 = goal setting (behaviour); 1.2 = problem solving; 1.4 = action planning; 1.5 = review behaviour goal(s); 1.6 = discrepancy between current behaviour and goal; 2.2 = feedback on behaviour; 2.3 = self-monitoring of behaviour; 2.4 = self-monitoring of outcome(s) of behaviour; 3.1 = social support (unspecified); 4.1 = instruction on how to perform the behaviour; 4.3 = re-attribution; 5.1 = information about health consequences; 5.6 = information about emotional consequences; 8.1 = behavioural practice/rehearsal; 8.6 = generalisation of target behaviour; 8.7 = graded tasks; 9.1 = credible source*

The resulting intervention consisted of seven face-to-face group discussion sessions and practical PA, delivered by a therapist over 12 weeks. The frequency and duration of sessions were designed to allow for gradual withdrawal of therapist support within a structured environment. This aimed to optimise self-efficacy and autonomy for PA behaviour change and encourage self-management.

The intervention was designed to be delivered by a therapist with knowledge of RA fatigue, PA and behaviour change. Optimal group size was specified as between six and 10 patients, but this could vary depending on local circumstances. The research team considered this large enough to minimise a diminished group-learning effect if attrition occurred, yet small enough to enable sufficient individual attention and support and ensure patient safety.

HCPs and patients emphasised the importance of choice and patient-led decision making to facilitate motivation and engagement in behaviour change. Self-determination theory [37] was selected as an underpinning theory for interactive delivery, using MI techniques such as open-ended questioning to encourage and facilitate patient-generated ideas. Self-determination theory distinguishes between two types of motivation: intrinsic (autonomous) and extrinsic (controlled) [37]. Autonomous motivation relates to a person’s sense of choice and personal importance when deciding to engage in behaviours such as PA, rather than taking part because someone has told them to (controlled motivation). They are more likely to spontaneously engage in a behaviour that satisfies their interest or enjoyment than one that they feel coerced into doing. Placing patients at the centre of decision-making regarding using PA to manage their fatigue is a central premise of this intervention.

#### 3d: development of session plans and support materials

An outline of intervention sessions is presented in Table 3. A guide was developed for each session to support delivery. Suggested questions and prompts were detailed for the main objectives for each session. Educational support materials were developed using ideas from other evidence based programmes [29–31]. Support materials were tested and modified by research team members. A list of equipment required to run the session was generated, including suggested exercises for the practical session. These were adapted, with permission, from a PA self-management intervention for chronic pain [30], and an upper limb self-management and exercise intervention for people with RA [38].

**Table 3:** Outline of session content

Week number	Session number	Group discussion topics (45–55 min) followed by coffee break (10–15 min)	Practical session (30–45 min)	Support materials and homework tasks
1	1	• Introduction to the course – aims and expectations, Ground rules and housekeeping	• Demonstration of exercises and gym equipment	• Handouts – Arthritis Research UK fatigue booklet, Causes of fatigue, PA in RA, List of exercises included in the practical session
		• Discussion topic: Share and discuss current feelings and experiences relating to fatigue and PA	• Patient choice of exercises with supervision as time allows	
				• Task – Activity diary to complete for next session
		• Discuss benefits of PA in RA		
		• Introduction to activity diaries		
2	2	• Review and discuss activity diaries	• Individual goal setting (PA goal)	• Handouts – Pacing, Graded approach to exercise, Goal setting, Borg scale
		• Activity analysis, pacing and energy management	• Discuss potential barriers to PA and possible solutions	
				• Task – Goal setting activity and graded PA plan, establish a baseline for chosen PA, continue activity diary
		• Introduce principles of graded approach to exercise and progression of PA	• Introduce Borg scale for monitoring exertion	
		• Introduction to goal setting	• Patient choice of exercises with supervision	
3	3	• Review and discuss pacing and activity analysis	• Review individual goals	• Handouts – sleep, stress and relaxation, relaxation CD
			• Patient choice of exercises with supervision	
		• Discuss impact of sleep and rest on PA and fatigue		• Task – try out relaxation CD, continue with PA goal and activity diary
			• Progression of exercises as appropriate	
		• Effects of stress and techniques for relaxation	• End with relaxation	
4	4	• Review general progress. Discuss barriers and potential solutions	• Review individual goals	• Handouts – self monitoring, pedometers, healthy diet
			• Patient choice of exercises with supervision	
		• Discuss ideas for self-monitoring PA		• Task – try out tools for self-monitoring and prompting PA, continue with PA goal and activity diary
			• Progression of exercises as appropriate	
		• Discuss diet and weight management in relation to PA		
6	5	• Review general progress	• Review individual goals	• Handouts –Managing external demands, managing setbacks
		• How to manage setbacks	• Patient choice of exercises with supervision	
				• Tasks – think about and formulate a setback plan, continue with PA goal and activity diary
		• Discuss managing PA alongside occupation		
			• Progression of exercises as appropriate	
8	6	• Review general progress	• Review individual goals	• Handouts – Template for planning long-term PA
		• Review and discuss setback plan	• Patient choice of exercises with supervision	
		• Discuss how to continue and maintain PA in the longer term		• Task – continue with PA goal and activity diary, complete long-term PA plan
			• Progression of exercises as appropriate	
12	7	• Review general progress, setback plan, options for long-term maintenance and continued progression of PA	• Review individual goals	• Handouts – List of resources for long-term PA
			• Patient choice of exercises with supervision	
			• Progression of exercises as appropriate	

*PA = physical activity; RA = rheumatoid arthritis*

## Discussion

This paper provides a detailed description of the development and co-design of a group-based RA fatigue self-management intervention based on modification of PA. Intervention development was based on MRC guidance for complex intervention development, and informed by contemporary behaviour change methodologies, including the BCW, TDF and BCT taxonomy. The resulting intervention aims to enable RA patients to develop their capability to self-manage fatigue through modifying PA, identify physical and social opportunities to support PA modification, and enhance motivation to modify PA within the context of their fatigue. The benefits of PA for managing fatigue in RA and other long-term conditions were identified from existing literature (phase 1a), and evidence of its use in clinical practice was provided by HCPs (phase 1b) and supported by participants (phase 2).

To our knowledge, this is the first study to provide evidence of RA patient and rheumatology HCP involvement, including extensive input from PRPs, in the co-design of a PA fatigue management intervention [34]. Consideration of implementation and participation issues and preferences of potential intervention users and those who might deliver it during development means that the intervention is likely to be acceptable to future users, and to translate into clinical settings [39].

Participant preferences influenced intervention design in several ways. For example, RA patients and HCPs indicated a desire for a face-to-face group programme, to provide an opportunity for shared experiences and vicarious learning. This finding suggests that other recent PA interventions for RA fatigue delivered on an individual basis [40, 41] may not adequately meet the needs of some RA patients. Whilst it is acknowledged that individual interventions may suit patients who do not like groups, results from the current study suggest that people with RA value social support that may be important for facilitating behaviour change. Group structures may facilitate formation of social networks which have been shown to influence health status and health-promoting behaviours, such as PA [42]. In addition, phase 2b HCPs reported that group delivery was consistent with current rheumatology practice, and potentially cost-effective, although this needs to be determined in future research.

Participant preferences determined the style of intervention delivery. The importance of choice and patient-led decision-making to facilitate motivation and engagement in behaviour change was emphasised by all participants, and use of MI techniques was recommended by HCPs. Placing patients at the centre of decision-making and enhancing autonomy regarding modification of PA to manage fatigue is a fundamental principle of this intervention. Interactive delivery processes were enhanced by selection of self-determination theory as an underpinning motivational theory. Person-centred approaches have been advocated for behaviour change interventions in musculoskeletal conditions [43] and have been successfully applied to promote PA in RA [44].

Recent PA interventions for RA fatigue have focused on individual home-based PA [40, 41, 45]. In the current intervention, inclusion of an education session and practical PA were important components. Patients wanted the opportunity to develop knowledge and practice using skills to enhance their capability to perform PA within a supportive environment. The group setting allows modelling of the behaviour by the group facilitator and other participants, and provides an opportunity for immediate feedback to facilitate behaviour change.

Challenges identified for implementation and service provision included location, timing and duration of sessions. Participants emphasised these must be suitable for patients, whilst taking into account local staffing and resource constraints. Key challenges included accessibility for people who work or who have other roles and responsibilities that prohibit attendance during the week. Rheumatology HCPs believed that flexible staffing arrangements may be possible within current services, but some patients may still not be able to attend face-to-face sessions. Future intervention development should explore alternative modes of delivery for these patients. For example, research has suggested that RA patients would be prepared to take part in internet-based interventions [46], suggesting this format may offer a viable alternative, whilst recognising that this removes the important supportive group environment.

### Strengths and limitations

The strength of the intervention development process described here lies in its thorough, systematic approach to design, and the extensive consideration of the needs and preferences of those who may use or implement the intervention in real-world settings. In addition, use of the BCW combined with the TDF and BCT taxonomy provided a comprehensive, structured method for developing a behaviour change intervention. Use of contemporary theories and models deepens our understanding of mechanisms by which interventions might bring about behaviour change, and facilitates comparison with similar interventions through use of standardised terminology.

Several limitations should be noted. Small numbers of patients and HCPs were involved in development. All participants in phase 2 voluntarily took part in focus groups, suggesting that they were predisposed to finding group settings acceptable. The views of those who do not like groups were not presented. Despite use of purposive sampling techniques, participants were a homogenous ethnic group, predominantly Caucasian, and able to speak English fluently. Data regarding socio-economic status were not recorded. Future intervention development should seek to understand the preferences and needs of a wider range of socio-cultural perspectives to ensure relevance and acceptability to a diverse population. Those responsible for commissioning services should also be involved, given their significant role in determining service implementation.

### Implications and future research

Further feasibility testing is required prior to full scale evaluation in order to understand whether the current intervention and implementation suggestions are appropriate, acceptable and possible to deliver in practice. Future development must consider how much flexibility in implementation and delivery is required and permissible to accommodate local context and resources without excessively diluting potential intervention effectiveness. Other adaptations should identify different delivery models for those who cannot or do not wish to attend groups or face-to-face programmes, address cultural contextual issues, and consider suitability for other inflammatory arthritis populations who experience fatigue.

## Conclusion

The MRC guidance and BCW provided a systematic process for developing a comprehensive, evidence-informed group self-management intervention for managing RA fatigue through modifying PA. This preliminary stage of development was important to ensure explicit links between the underpinning theory, mediating pathways and intervention outcomes thus facilitating future implementation and evaluation. Consultation and collaboration with potential recipients and professionals who might deliver the intervention enhances the likelihood of acceptability and implementation in future clinical practice. The feasibility of further evaluation now needs to be determined by further investigation of acceptability, implementation and practicality of the intervention.

## Abbreviations

BCT: Behavior change technique
CFS: Chronic fatigue syndrome
COM-B: Capability, Opportunity, Motivation – Behaviour
HCP: Healthcare professional
MI: Motivational interviewing
MRC: Medical Research Council
NICE: National Institute for Health and Care Excellence
PA: Physical activity
PRP: Patient research partner
RA: Rheumatoid arthritis
TDF: Theoretical Domains Framework
